# Monitoring mortality in the setting of COVID-19 pandemic control in Victoria, Australia: a time series analysis of population data

**DOI:** 10.5365/wpsar.2025.16.01.1091

**Published:** 2025-01-16

**Authors:** Lalitha Sundaresan, Sheena G Sullivan, David J Muscatello, Daneeta Hennessy, Stacey L Rowe

**Affiliations:** a Mathematica, Oakland, California, United States of America.; b WHO Collaborating Centre for Reference and Research on Influenza, Royal Melbourne Hospital, Melbourne, Victoria, Australia.; c Department of Epidemiology, University of California Los Angeles, Los Angeles, California, United States of America.; d Public Health Division, Department of Health, Melbourne, Victoria, Australia.; e School of Population Health, University of New South Wales, Sydney, New South Wales, Australia.; f School of Population Health and Preventive Medicine, Monash University, Melbourne, Victoria, Australia.; * These authors contributed equally.

## Abstract

**Objective:**

Mortality surveillance was established in the state of Victoria just before the COVID-19 pandemic. Here, we describe the establishment of this surveillance system, justify the modelling approach selected, and provide examples of how the interpretation of changes in mortality rates during the pandemic was influenced by the model chosen.

**Methods:**

Registered deaths occurring in Victoria from 1 January 2015 to 31 December 2020 were sourced from the Victoria Death Index. Observed mortality rates were compared to a raw historical 5-year mean and to predicted means estimated from a seasonal robust regression. Differences between the observed mortality rate and the historical mean (∆MR) and excess mortality rate from the observed and predicted rates were assessed.

**Results:**

There were 20 375 COVID-19 cases notified in Victoria as of 31 December 2020, of whom 748 (3.7%) died. Victorians aged ≥ 85 years experienced the highest case fatality ratio (34%). Mean observed mortality rates in 2020 (MR: 11.6; 95% confidence interval [CI]: 11.4, 11.9) were slightly reduced when compared with the annual rate expected using the historical mean method (mean MR: 12.2; 95% CI: 12.1–12.3; ∆MR: −0.57; 95% CI: −0.77, −0.38), but not from the rate expected using the robust regression (estimated MR: 11.7; 95% prediction interval [PI]: 11.5, 11.9; EMR: −0.05; 95% CI: −0.26, 0.16). The two methods yielded opposing interpretations for some causes, including cardiovascular and cancer mortality.

**Discussion:**

Interpretation of how pandemic restrictions impacted mortality in Victoria in 2020 is influenced by the method of estimation. Time-series approaches are preferential because they account for population trends in mortality over time.

Mortality surveillance is widely used to understand and forecast trends and patterns of mortality over time, thus guiding the development of policy to reduce the burden of specific causes of disease and death. (*1*, *2*) Specific applications include monitoring the health impacts of significant public health events, such as extreme temperatures, (*3*) bushfires (*4*, *5*) and epidemics. (*6*, *7*) In 2020, existing surveillance systems proved a useful tool for monitoring the direct and indirect impact of the COVID-19 pandemic on mortality. (*8*, *9*) Notably, in countries with limited circulation of SARS-CoV-2, mortality was lower than expected in 2020, (*10*) while in countries with large epidemics, mortality was in excess. (*8*)

Different approaches to monitoring mortality have been implemented during the pandemic. (*11*) One simple method, employed by some countries, (*10*, *12*, *13*) compares mean mortality for some historical period with current-year rates. While easy to implement, this approach does not accommodate time trends in expected mortality, which generally declines over time, consistent with increasing life expectancy. Time-series regression models overcome this problem by incorporating parameters that predict seasonal mortality patterns and predict the increased mortality typically observed during winter months. (*7*) Various regression approaches have been adopted by national surveillance systems for estimating excess COVID-19-attributable mortality and mortality rates (MRs). (*11*, *14*, *15*) While there are many regression model options available, comparison of different modelling options for influenza surveillance suggests they yield similar estimates. (*7*, *16*, *17*) Moreover, real-time surveillance data availability may be delayed, making some of these approaches inappropriate.

In 2019, mortality surveillance was newly established in the state of Victoria in anticipation of the seasonal influenza epidemic. This surveillance was rapidly adapted in early 2020 to enable real-time situation assessment of changes in mortality associated with COVID-19 infections and restrictions. Here, we describe mortality surveillance in Victoria, provide a summary of COVID-19 deaths in 2020, and compare two methods for real-time monitoring of mortality for public health decision-making.

## Methods

### Data sources

All laboratory-confirmed cases of COVID-19 are notifiable to the Victorian Department of Health (the Department) under public health and well-being legislation. These data were sourced from the Public Health Event Surveillance System (PHESS), along with demographic, clinical and epidemiological risk information. All notified deaths of people with COVID-19 were recorded in this system when official notifications were made to the Department or during case or outbreak follow up. Since these data capture individuals who died due to COVID-19 as well as other causes, deaths where this distinction was unclear underwent clerical review by a multidisciplinary team of public health and infectious disease medical practitioners and epidemiologists in accordance with the national case definition. (*18*)

Data for all registered deaths between 2015 and 2020 were sourced from the Victorian Death Index (VDI), maintained by the Registrar of Births, Deaths and Marriages. The data provided consisted of an electronic copy of the medical certificate of cause of death for all deaths registered in Victoria, including coroner-referred deaths. The certificate included free-text fields for direct, antecedent and other causes of death, as well as other information such as age, sex, date of birth, date of death, marital status, parents’ details, number of siblings, number and age of children and address of the deceased.

Cause-specific deaths were identified using keyword searches in the free-text causes of death fields in the VDI data using the multiple causes of death methodology (see **Supplementary Information**). (*2*) The term “multiple causes of death” refers to all conditions listed in the death certificate. If the death certificate included any mention of a condition in any of the text fields of causes of death including the direct cause, antecedent cause or other causes, then that deceased person was categorized as having that specific cause of death as one of their causes of death. Data management was conducted using the Stata® statistical package version 16.



A stringency index, categorized into five levels, was developed based on key restrictions implemented by the state government (see **Supplementary Information**). (*2*) These included restrictions on mobility, social and religious congregation, school and workplace attendance, health and aged-care facility visitation, and access to dining, retail and services. Restrictions were initially implemented in March–April 2020, relaxed in May 2020 with successful containment of SARS-CoV-2 transmission, and then reimplemented in July–October 2020 when new cases were detected due to transmission to workers in hotel quarantine. The implementation and categorization of these restrictions have been described in detail elsewhere. (*19*)

Population denominators for the calculation of MRs were derived from mid-year resident population estimates provided by the Australian Bureau of Statistics for 2015–2019 (*20*) and from the Victorian Department of Environment, Land, Water and Planning for 2020. (*21*)

### Statistical analysis

COVID-19 incidence rates, case fatality ratios (CFR) and MRs were calculated overall and for pre-defined age groups (< 65 years, 65–74 years, 75–84 years, ≥ 85 years). The incidence of COVID-19 for 2020 was calculated as the number of notified COVID-19 cases per 100 000 population. CFRs were calculated as the proportion of notified COVID-19 deaths among all notified COVID-19 cases. The COVID-19 MR was calculated as the number of COVID-19 deaths among the total population.

The weekly MR was calculated as the weekly number of deaths divided by the population for each age group and cause of death and converted to a rate per 100 000 population. The specific causes of interest included pneumonia and influenza, respiratory causes, cardiovascular disease, cancer and injuries including accidents.

Two methods for assessing the deviation in MRs were used. In the first, the observed weekly MR was compared with the historical mean weekly MR based on the prior 5 years’ data (2015–2019). Differences in the weekly observed and historical mean rates were calculated and averaged to estimate the annual mortality rate difference (∆MR).

Second, excess mortality was estimated as the difference between the observed weekly MR and the expected weekly MR predicted from a seasonal robust linear regression model fit using the observed weekly MRs in the previous 5 years. Data were fit using the rlm function in the MASS package in R (see **Supplementary Information** for associated R scripts), assuming the following equation:

This method is an extension of the well established Serfling method, (*22*, *23*) and incorporates a sinusoidal term to predict the seasonal trend in mortality typically observed in temperate settings. Standard errors were estimated using Tukey’s bisquare function, which is robust to outliers. (*24*) This approach was chosen over other options, such as a Poisson regression, to align with methods used in national surveillance (*25*) and in other states, (*23*) and because prior studies using Australian data had reported minimal differences in overall estimates using different approaches. (*16*)

The excess mortality rate (EMR) was estimated as the weekly observed MR minus the predicted MR from the model. We refer to it as the excess MR, even where the estimates were negative, suggesting lower-than-expected MRs. The epidemic threshold that differentiates extreme mortality events (both epidemics and periods of lower-than-expected mortality) from random variation was set as follows:

Deviations in observed MRs for 2020 from expected MRs based on either the historical mean or the seasonal robust regression estimates were visually assessed with respect to the stringency of restrictions in place at different times during 2020.

## Results

### COVID-19 deaths

From 27 March to 31 December 2020, there were 20 375 COVID-19 cases in Victoria, Australia. These cases were associated with 748 registered deaths attributed to COVID-19, with another 72 deaths being attributed to other causes. Deaths arising from COVID-19 in Victoria were not evenly distributed across age groups and were positively correlated with age (**Table 1**). People aged < 65 years accounted for 84% of COVID-19 cases, with an incidence rate (IR) of 302 per 100 000 population. However, deaths in this age group were low (CFR: 0.2%; MR: 0.46 per 100 000 population). In contrast, Victorians aged ≥ 85 years comprised just 6.6% of notified COVID-19 cases but experienced far higher fatality and MR (CFR: 34%, MR: 323 per 100 000 population).

**Table 1:** COVID-19 confirmed cases, incidence rates, registered deaths, case fatality ratio and mortality rates, Victoria, Australia, 2020

Age group (years)	Cases	Distribution (%)	Population	Incidence rate (per 100 000 population)	Deaths	Case fatality ratio (%)^a^	COVID-19 mortality rate (per 100 000 population)^b^
** < 65**	**17 124**	**84.0**	**5 678 949**	**302**	**26**	**0.2**	**0.5**
**65–74**	**970**	**4.8**	**582 720**	**166**	**62**	**6.4**	**11**
**75–84**	**945**	**4.6**	**328 475**	**288**	**209**	**22.1**	**64**
** ≥ 85**	**1336**	**6.6**	**139 481**	**958**	**451**	**33.8**	**323**
**Total**	**20 375**	**100**	**6 729 626**	**302**	**748**	**3.7**	**11**

^a^ The case fatality ratio is calculated as the number of COVID-19 deaths among all notified COVID-19 cases.

^b^ The COVID-19 mortality rate is calculated as the number of COVID-19 deaths among the total population.

### Mortality and COVID-19 restrictions

**Fig. 1:**
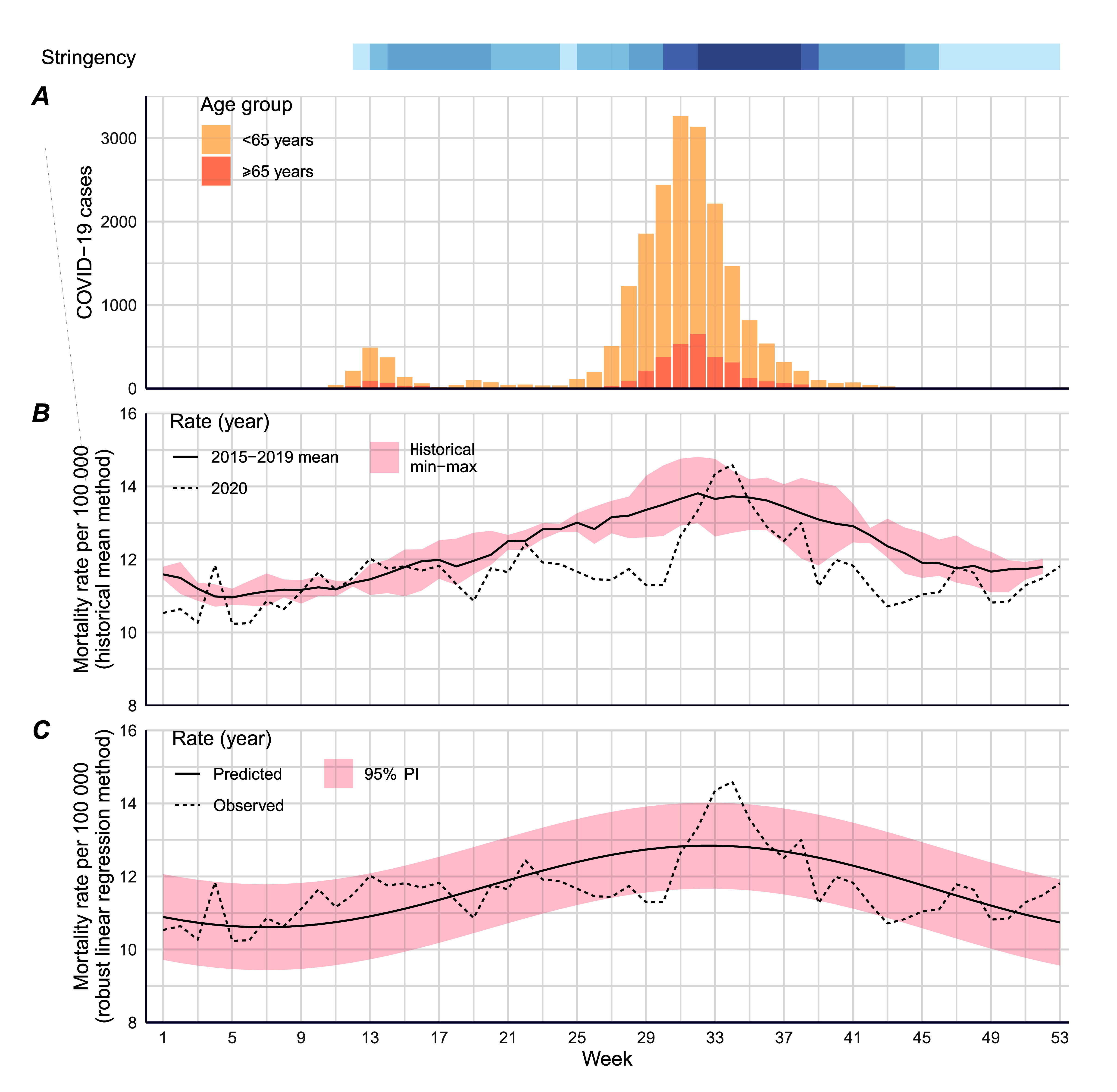
Weekly all-cause mortality in Victoria, Australia, considering COVID-19 notifications and pandemic restrictions

Weekly MRs observed in 2020 against COVID-19 cases and pandemic restrictions are shown in **Fig. 1 (B, C)**. Stage 1 restrictions were introduced in mid-March 2020 and were rapidly ramped up to stay-at-home orders (Stage 3) at the end of March (week 14). Mortality rates declined coincident with Stage 3 restrictions and dropped by week 19. Rates did not appreciably increase again until week 31, at the height of the second epidemic wave, which was characterized by a series of outbreaks in residential aged-care facilities. (*19*, *26*) Mortality rates peaked when restrictions were most stringent, consistent with efforts to limit SARS-CoV-2 transmission and the peak in case fatality among residents in aged-care settings. The control of the SARS-CoV-2 epidemic and relaxation of restrictions were followed by a return to MRs lower than the historical mean and estimated rates.

[**insert** Figure 1]

### Mean mortality rate difference based on the historical mean

All-cause weekly MRs observed in 2020 compared with the 5-year historical mean are shown in **Fig. 1B**. The all-cause MR in 2020 (MR: 11.6; 95% confidence interval [CI]: 11.4, 11.9) was lower than the historical mean (MR: 12.2; 95% CI: 12.1–12.3), with a mean difference (∆MR) of −0.57 (95% CI: −0.77, −0.38), representing a modest decrease. This trend of reduced mortality in 2020 was replicated in all age groups examined (**Table 2**), with the greatest difference observed for those aged ≥ 85 years (∆MR: −11.78; 95% CI: −17, −6.6).

**Table 2:** Summary of mean observed (2020) versus 5-year mean (2015–2019) all-cause mortality rate per 100 000 population, by age group, Victoria, Australia

Age group (years)	Observed mortality rate 2020 (95% CI)	Historical mean mortality rate 2015–2019 (95% CI)	Mean rate difference (95% CI)	Predicted mortality rate (95% PI)	Excess mortality rate (95% CI)
** < 65**	**2.28 (2.22, 2.35)**	**2.43 (2.40, 2.46)**	**-0.15 (−0.22, −0.07)**	**2.33 (2.32, 2.35)**	**-0.05 (−0.11, 0.02)**
**65–74**	**20.5 (19.9, 21.0)**	**21.6 (21.3, 21.9)**	**-1.13 (−1.8, −0.48)**	**20.89 (20.60, 21.19)**	**-0.44 (−1.03, 0.15)**
**75–84**	**60.7 (59.0, 62.5)**	**67.1 (66.2, 68.1)**	**-6.43 (−7.9, −5.0)**	**60.9 (59.73, 62.09)**	**-0.13 (−1.66, 1.40)**
** ≥ 85**	**240 (232, 248)**	**252 (248, 256)**	**-11.78 (−17, −6.6)**	**240 (233.15, 245.93)**	**0.61 (−5.16, 6.38)**

CI: confidence interval; PI: prediction interval.

Week 53 is removed for comparisons. Predicted rates are estimated from the robust linear regression model using weekly mortality data during 2015–2019.

A similar trend of lower observed mortality than that expected based on the historical mean was noted for each of the cause-specific MRs (**Table 3**). The observed MR in 2020 was lower than the historical mean for pneumonia and influenza (∆MR: −0.46; 95% CI: −0.55, −0.37), respiratory (∆MR: −0.71; 95% CI: −0.83, −0.58) and cardiovascular causes (∆MR: −0.58; 95% CI: −0.69, −0.48). More modest decreases in mortality were observed for cancer deaths, accidents and injuries (**Table 3**).

**Table 3:** Summary of mean observed (2020) versus 5-year mean (2015–2019) all-cause mortality rate per 100 000 population, by cause of death, Victoria, Australia

Cause of death	Observed mortality rate 2020 (95% CI)	Historical mean mortality rate 2015–2019 (95% CI)	Mean rate difference (95% CI)	Predicted mortality rate (95% PI)	Excess mortality rate (95% CI)
**All causes**	**11.6 (11.4, 11.9)**	**12.2 (12.1, 12.3)**	**-0.57 (−0.77, −0.38)**	**11.7 (11.5, 11.9)**	**-0.05 (−0.26, 0.16)**
**Pneumonia and influenza**	**1.14 (1.07, 1.20)**	**1.60 (1.55, 1.65)**	**-0.46 (−0.55, −0.37)**	**1.28 (1.21, 1.36)**	**-0.15 (−0.24, −0.07)**
**Respiratory**	**2.77 (2.65, 2.89)**	**3.48 (3.39, 3.56)**	**-0.71 (−0.83, −0.58)**	**2.78 (2.66, 2.91)**	**-0.02 (−0.15, 0.12)**
**Cardiovascular**	**5.03 (4.90, 5.15)**	**5.61 (5.50, 5.72)**	**-0.58 (−0.69, −0.48)**	**4.45 (4.31, 4.58)**	**0.57 (0.47, 0.68)**
**Cancer**	**3.77 (3.69, 3.84)**	**3.92 (3.87, 3.96)**	**-0.15 (−0.23, −0.07)**	**3.56 (3.54, 3.58)**	**0.21 (0.13, 0.28)**
**Injury**	**0.43 (0.40, 0.46)**	**0.57 (0.55, 0.58)**	**-0.14 (−0.17, −0.10)**	**0.47 (0.46, 0.47)**	**-0.03 (−0.06, 0.00)**

CI: confidence interval; PI: prediction interval.

Week 53 was removed for comparisons. Predicted rates are estimated from the robust linear regression model using weekly mortality data during 2015–2019.

### Excess mortality rate estimated from the seasonal robust regression model

**Fig. 2:**
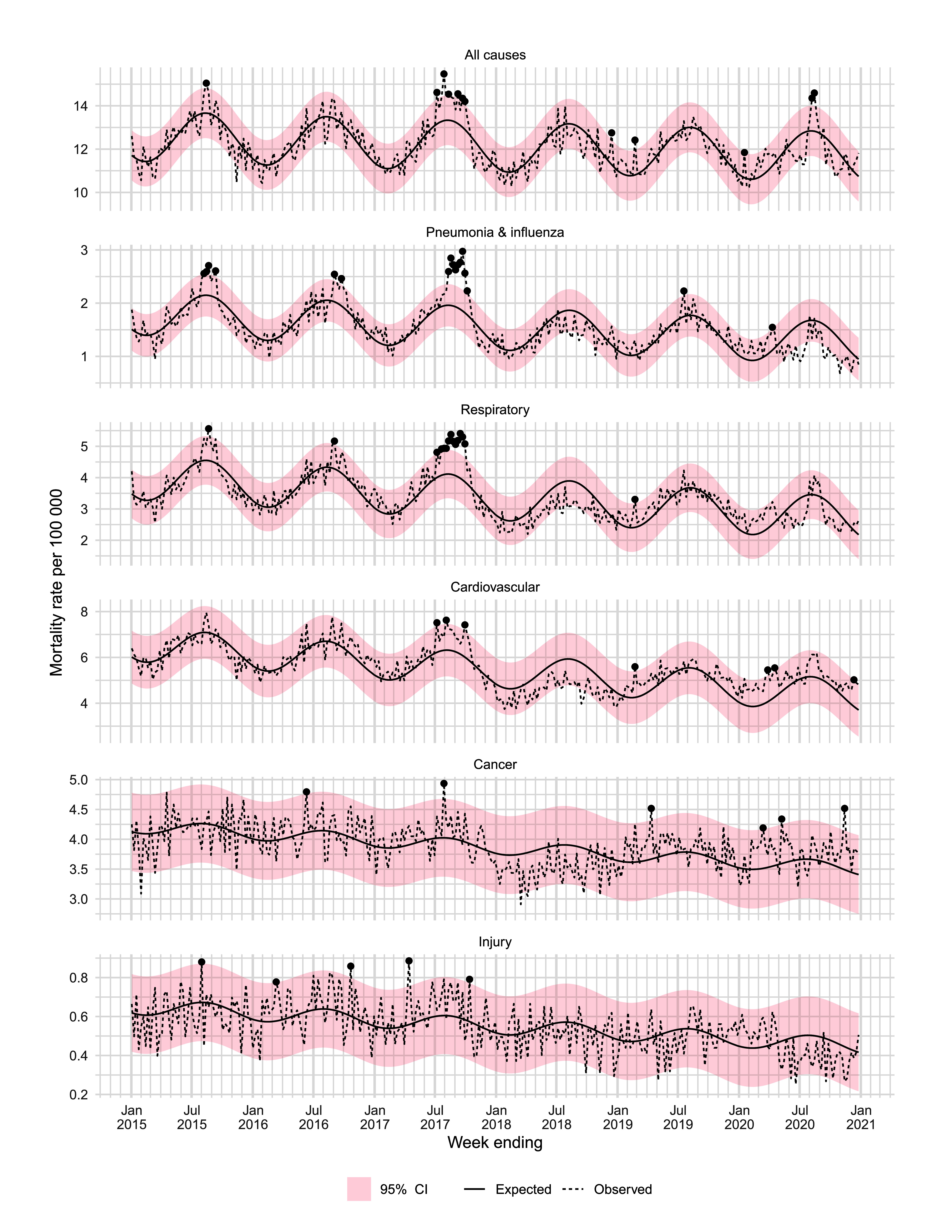
Weekly cause-specific mortality rates estimated from the robust regression model, Victoria, Australia, 2015–2020

As shown in **Fig. 1C**, weekly all-cause MRs observed in 2020 were both higher and lower than the estimated rates predicted by the seasonal robust regression model. However, the all-cause predicted mortality estimate was 11.7 (95% prediction interval [PI]: 11.5, 11.9), which was comparable with the observed rate (PI: 11.6; 95% CI: 11.4–11.9), and there was thus a negligible net difference across the year with an EMR of −0.05 (95% CI: −0.11, 0.02; **Table 2**). Predicted all-cause mortality over the entire estimation period is shown in **Fig. 2**. Notable weeks of excess mortality can be seen in 2017, during which there was a severe influenza epidemic (*27*) (**Fig. 2**).

[**insert** Figure 2]

By age, observed MRs were lower than the estimated rates expected from the model for most age groups, except for those aged ≥ 85 years, for whom the rate was slightly higher, albeit with wide CIs (EMR: 0.61; 95% CI: −5.16, 6.38). Unlike the estimates using the historical mean, the CIs around the age-specific estimates of excess mortality included 0, suggesting the results were compatible with either an increase or decrease in mortality (**Table 2**).

Cause-specific estimates of excess mortality are shown in **Table 3**, and the modelled weekly estimates are shown in **Fig. 2**. Seasonality trends were most apparent for pneumonia and influenza, respiratory and cardiovascular causes (**Fig. 2**). There was a decline in mortality from pneumonia and influenza (EMR: −0.15; 95% CI: −0.24, −0.07), consistent with the analysis using the historical mean. However, unlike the historical mean method, observed mortality in 2020 was estimated to be higher than expected for cardiovascular (EMR: 0.57; 95% CI: 0.47–0.68) and cancer causes (EMR: 0.21; 95% CI: 0.13–0.28).

## Discussion

The direct global mortality burden of COVID-19 has without a doubt been substantial. (*28*) However, the extent to which this may have been offset by pandemic mitigation measures deserves attention. To this end, we explored the impact of COVID-19 and associated containment measures on mortality dynamics in Victoria. Despite the substantial direct mortality burden attributable to COVID-19, there was no overall excess mortality in Victoria during the first year of the COVID-19 pandemic, 2020, highlighting the countervailing impact of containment and mitigation measures.

Although we did not detect higher-than-expected net mortality across 2020, MRs did deviate from their expected values at various times, being both substantially higher and lower than expected at different stages of the pandemic. During the first epidemic wave, a small spike in mortality was observed, followed by a drop when mitigation measures were initially introduced. Observations of reduced mortality during periods of pandemic restrictions have also been reported internationally. In New Zealand, border closures and strict mitigation measures early in the pandemic successfully limited SARS-CoV-2 circulation (*29*) and were associated with reduced all-cause (notably pneumonia-influenza) mortality. (*10*) Our findings also accord with an early pandemic study (February–May 2020), which examined all-cause mortality in 21 industrialized countries and showed that Australia was one of the few countries that avoided a detectable rise in all-cause mortality during the first wave of the COVID-19 pandemic, having the seventh-lowest level of excess deaths among the surveyed countries. (*28*)

As the pandemic progressed, a much larger spike in mortality was observed during Victoria’s second epidemic wave, leading to substantial COVID-19 excess mortality, which offset the reductions in mortality seen in earlier months. The CFR in Victoria was high at 3.9%, reaching up to 34% in the oldest age groups, reflecting the large number of outbreaks in residential aged-care facilities. Residents of aged-care facilities comprised 10% of all COVID-19 cases in Victoria in 2020 and 80% of all deaths. (*19*) Other countries, such as Japan and Singapore, which had comparable COVID-19 incidence rates but far lower CFRs at the time, (*30*) were successful in preventing outbreaks in residential aged-care facilities.

A key outcome of our study was the discrepancy in estimated excess mortality that was observed when we used different methods to measure the expected weekly mortality rate. We used both a simple historical mean method and a seasonal robust linear regression. The former approach compares a mean mortality rate without adjustment for trend associated with changes in life expectancy. (*31*) In our study, this method overestimated excess mortality, but in other settings it may underestimate excess mortality. (*11*) The robust linear regression predicted a lower weekly mortality rate in 2020 because mortality in the years used for estimation (2015–2019) was steadily decreasing, and the model predicted this trend to continue. As a result, differences in observed and expected mortality were less pronounced using the regression approach compared with the 5-year historical mean approach. This has implications for interpretation of national data, and those countries that reported estimated mortality during the pandemic against the historical mean may have over- or underestimated the true rate.

Within our own data, the difference between these methods could lead to different interpretations of the pandemic’s impact on mortality. For example, pneumonia and influenza mortality rates were lower than expected using both methods. This effect was probably quite large in most settings and can be attributed to disruptions to usual seasonal activity of respiratory pathogens, most notably influenza and respiratory syncytial virus, due to containment measures enacted in response to the pandemic, for which there is substantial evidence both locally (*32*-*34*) and globally. (*35*, *36*) In contrast, the robust linear regression analysis indicated that cardiovascular and cancer mortality rates were higher than expected, while the comparison to the historical mean suggested that rates for these causes were lower than expected.

Reasonable explanations for both an increase and a decrease might be possible. A potential hypothesis for excess mortality due to cardiovascular and cancer causes is that stay-at-home orders may have led to a delay in screening and seeking medical treatment for noncommunicable diseases. (*37*, *38*) In contrast, the observation of increased (as opposed to decreased) cardiovascular mortality runs counter to prior observations that have associated excess cardiovascular mortality with influenza infection, and influenza all but disappeared during the study period. (*39*, *40*) Further investigation is required to disentangle this paradox. Nevertheless, our assertion is that estimates of excess mortality do need to account for the time trend, in which case, the seasonal robust regression is expected to provide a more reliable estimate of mortality.

One of the major strengths of this mortality surveillance is the utilization of near-real-time death data. To expedite real-time reporting in 2020, we did not code the free-text causes of death into International Classification of Disease 10 (Australian Modification) codes, but instead used keyword search terms, an approach successfully implemented in the neighbouring state of New South Wales for many years. (*23*) Moreover, we used multiple cause of death methodology, rather than using the principal cause of death. National mortality surveillance conducted by the Australian Bureau of Statistics standardizes the cause of death from data provided by death registries in each Australian state and territory using the World Health Organization *International Classification of Diseases, Tenth Revision (ICD-10)* codes. That surveillance approach is slower because of the need for coding. Moreover, only the underlying cause of death is considered (the disease or injury that initiated the chain of morbid events leading directly to death), which may lead to differences in the number of deaths counted for each cause. This discordance in methodology is a potential limitation of our work in that it makes it challenging to compare our results with other published estimates from Australia. (*25*) Nevertheless, the use of seasonal robust linear regression and the number of prior years’ data used for estimation are similar, and the overall pattern of mortality observed in our study did not substantially deviate from the ICD-coded data analysed by the Australian Bureau of Statistics. (*25*)

Our study only assessed mortality dynamics during 2020. This allowed us to focus on excess mortality at the time this surveillance system was set up in Victoria and to evaluate two options for conducting that surveillance. Further work could examine in more detail how mortality continued to evolve throughout the pandemic, for example, to explore whether there was any displaced mortality (sometimes referred to as “harvesting”) associated with deaths prevented in 2020, or the role of vaccination and public health and social measures. Furthermore, a key purpose of this paper is to show that the measurement of excess mortality is quite a subtle concept and that it is sensitive to the manner in which expected mortality is measured. While this can be construed as a limitation in that it precludes the identification of a single, clear-cut measure of excess mortality, we view this as a methodological contribution of our paper.

This paper provides an overview of COVID-19-associated and excess mortality in Victoria, Australia, during the first year of the COVID-19 pandemic. We observed no excess mortality in 2020; however, our determination of this depended on the method chosen. We have highlighted the limitations of simple methods to estimate excess mortality and the need to consider long-term trends. Regardless of which method is most correct, given the high risk of all-cause, pneumonia and influenza and COVID-19 mortality for those in older age groups, efforts to limit the introduction and spread of disease in communities of older individuals need continued and sustained attention. Above all, this paper highlights the value of mortality surveillance in providing timely intelligence relating to the impact of major public health events on local populations, which can inform the design and implementation of mitigation and containment measures.
