# Supplementary material for: Monitoring mortality in the setting of COVID-19 pandemic control in Victoria, Australia: a time series analysis of population data
Source: Western Pac Surveill Response J. 2025 Jan 16;16(1):1–11. doi: 10.5365/wpsar.2025.16.01.1091 (PMC11855116; doi:10.5365/wpsar.2025.16.01.1091)
Supplement: Supplementary file 1 [file wpsar-16-1091-s001.pdf]

SUPPLEMENTARY INFORMATION

1. List of keyword searches used in the categorization of medically certified causes of death

Cause of death	Inclusions	Exclusions
Confirmed COVID-19	CORONAVIRUS, CORONAVIRUS DISEASE 2019, 2019-NCOV, CORUNAVIRUS, CORONA VIRUS, NOVEL CORONAVIRUS, SARS CORONAVIRUS, SARS-COV-2, SARSCOV2, SARSCOV, CORONAVIRUS2, COVID, COVID-19, COVID-19 POSITIVE TEST	
Unconfirmed COVID-19	SUSPECTED CORONAVIRUS DISEASE 2019, POSSIBLY CORONAVIRUS DISEASE 2019, POSSIBLE CORONAVIRUS DISEASE 2019, LIKELY CORONAVIRUS DISEASE 2019, NEGATIVE FOR SEVERE ACUTE RESPIRATORY SYNDROME CORONAVIRUS 2, COVID-19 NEGATIVE, CORONAVIRUS DISEASE 2019 NEGATIVE, SEVERE ACUTE RESPIRATORY SYNDROME CORONAVIRUS 2 NEGATIVE, N.E.C CORONAVIRUS	
Influenza	INFLUENZA, INFULENZA, INFLEUNZA, INFUENZA, FLU INFECTION, H1N1, HINI VIRAL, SWINEFLU, SWINE INFLU, SWINE FLU, SWINE INF, HAEMOPHILUS INFLUENZAE AND INFLUENZA A	HAEMOPHILUS INFLUENZAE, HAEMOPHILLUS INFLUENZAE, HEMOPHILUS INFLUENZAE, HEAMOPHILUS INFLUENZAE, HAEMOPHILUS INFLUENZA, HAEMOPHILLUS INFLUENZA, HAEMOPHILUSINFLUENZAE, HAEMOPHILLUSINFLUENZAE, HAEMOPHILUSINFLUENZA, HAEMOPHILUS INFULENZAE, HAEMOPHILLUS INFULENZAE, HAEMOPHILUS INFULENZA, HAEMOPHILLUS INFULENZA, HAEMOPHILUSINFULENZAE, HAEMOPHILLUSINFULENZAE, HAEMOPHILUSINFULENZA, HAEMOPHILLUSINFULENZA, HEMOPHILIS INFLUENZA, HEMOPHILUS INFLUENZA, HEMOPHILLUS INFLUENZA, HAEMOPHILIUS INFLUENZAE, HEMOPHYLLIS INFLUENZA, H INFLUENZAE, PARA-INFLUENZAE, PARAINFLUENZA, PARA INFLUENZA, PARA INFULENZA, PARA-INFLUENZA, PARAINFULENZA
Pneumonia	PNEUMONIA, PNEUEMONIA, PNEMONIA, PNEOMONIA, PENUMONIA, PNUEMONIA, PHEUMONIA, PNEUMONAL, PNEUMONA, PNEUMONIE, PNEUMONNIA, PNEUMONITIS, PNEUMONITITIS, LOWER RESPIRATORY TRACT INFECTION, SEVERE PNEUMONIA, REFRACTORY PNEUMONIA, NASOPHARYNGEAL ASPIRATE, PNEUMONIA WITH ASSOCIATED SEPSIS, COMMUNITY ACQUIRED LEFT LOWER LOBE PNEUMONIA, LEFT LOWER LOBE COMMUNITY ACQUIRED PNEUMONIA, BILATERAL STREPTOCOCCAL PNEUMONIA	ASPIRATION, ASPIRATE, ASPIRANION, ASPIRATION PNEUMONIA, HYPERSENSITIVITY PNEUMONITIS, RADIATION PNEUMONITIS, INTERSTITIAL PNEUMONITIS, LEFLUNOMIDE PNEUMONITIS, CHLORAMBUCIL PNEUMONITIS, BISULPHAN INDUCED PNEUMONITIS, ASPIRATION PNEUMONITIS, STREPTOCOCCUS PNEUMONIAE SEPSIS, CHEMICAL PNEUMONITIS, SEVERE PNEUMONIA (ASPIRATION), SEVERE PNEUMONIA ASPIRATION
Injuries	HEAD INJURY, FRACTURED NECK OF FEMUR, BLUNT HEAD TRAUMA, HEAD INJURIES, FRACTURED RIGHT NECK OF FEMUR, FRACTURED LEFT NECK OF FEMUR, ELECTROCUTION, INJURIES SUSTAINED, SUBDURAL HAEMATOMA, CHEST INJURIES, HEAD AND NECK INJURY, SUBDURAL HAEMORRHAGE, SUSTAINED IN A FALL, INJURY SUSTAINED, FOLLOWING A FALL, INJURIES RECEIVED, HEAD AND CHEST INJURIES, TRAUMATIC SUBDURAL HEMATOMA, ABDOMINAL INJURIES, ACQUIRED BRAIN INJURY, INCISED INJURIES, TRAUMATIC AMPUTATION, MULTIPLE RIB FRACTURES, BLUNT FORCE TRAUMA, IN A FALL, MULTITRAUMA, FRACTURE, OF A FALL, INCISED INJURY, PEDESTRIAN, DRIVER, PASSENGER, PEDAL CYCLIST, OCCUPANT, MOTORCYCLE, MOTOR VEHICLE, TRAUMATIC SUBARACHNOID HAEMORRHAGE, CRUSH INJURY	

Cause of death	Inclusions	Exclusions
Cardiovascular diseases	MYOCARDIAL, PERICARDIAL, RHEUMATIC, RHUEMATIC, MITRAL AORTIC, TRICUSPID, VALVE, VALVULAR, INTRAVASCULAR, HEART, CONGESTIVE, CORONARY, CARDIAC, CARDIO, DRESSLER'S SYNDROME, ANGINA, RUPTURE OF CHORDA TENDINEAE, RUPTURE OF PAPILLARY MUSCLE, ATHEROSCLEROTIC, ATHEROSCLEROSIS, ANEURYSM OF HEART, CORONARY ARTERY ANEURYSM, CARDIOMYOPATHY, PULMONARY EMBOLISM, PULMONARY EMBOLUS, PULMONARY HYPERTENSION, MYOCARDITIS, PERICARDITIS, ENDOCARDITIS, AORTITIS, CARDIOMEGALY, HAEMOPERICARDIUM, OESOPHAGEAL VARICES, VARICEAL BLEEDING, UPPER GASTROINTESTINAL BLEED, ATRIOVENTRICULAR BLOCK, INTRAVENTRICULAR BLOCK, FASCICULAR BLOCK, BUNDLE BRANCH BLOCK, CONDUCTION DISORDER, ATRIAL, VENTRICULAR, PREMATURE DEPOLARISATION, SICK SINUS SYNDROME, PULSELESS ELECTRICAL ACTIVITY ARREST, BRADYCARDIA, TACHYCARDIA, CEREBROVASCULAR, CEREBRO VASCULAR ACCIDENT, INTRACRANIAL HAEMORRHAGE, INTRACEREBRAL HAEMORRHAGE, SUBARACHNOID HAEMORRHAGE, SUBDURAL HAEMORRHAGE, EXTRADURAL HAEMORRHAGE, FRONTOPARIETAL HAEMORRHAGE, TEMPORAL INTRAPARENCHYMAL HAEMORRHAGE, CEREBRAL, CEREBELLAR, BASILAR, STROKE, TRANSIENT ISCHAEMIC ATTACK, BRAIN STEM INFARCT, BASAL GANGLIA INFARCTION, HYPERTENSIVE ENCEPHALOPATHY, VASCULAR LEUCOENCEPHALOPATHY, MOYAMOYA DISEASE, NONPYOGENIC THROMBOSIS OF INTRACRANIAL VENOUS SYSTEM, ANEURYSM, DISSECTION, HIGH BLOOD PRESSURE, HYPOTENSION, HYPERTENSION, ARTERIOVENOUS FISTULA, ARTERITIS, ARTERIAL, ARTERY, ARTERIOLE, PERIPHERAL VASCULAR DISEASE, THROMBOANGITIS OBLITERANS, RAYNAUD, PHLEBITIS, THROMBOPHLEBITIS, EMBOLISM, THROMBOSIS, SHOCK, LYMPHANGITIS, LYMPHADENITIS, LYMPHOEODEMA	
Respiratory diseases	BRONCHITIS, BRONCHIECTASIS, EMPHYSEMA, CHRONIC OBSTRUCTIVE, CHRONIC LUNG DISEASE, CHRONIC OBSTUCTIVE PULMONARY DISEASE, CHRONIC OBSTRUCTION PULMONARY DISEASE, EXACERBATION OF AIRWAYS DISEASE, COPD, COAD, OBSTRUCTIVE LUNG, CHRONIC AIRWAYS, AIRWAYS DISEASE, AIRWAY DISEASE, ASTHMA, STATUS ASTHMATICUS, RESPIRATORY, PNEUMONIA, PNUEMONIA, PNEMONIA, PNEOMONIA, PNEAMONIA, PENUMONIA, PNUEMONIA, PHEUMONIA, PNEUMONAL, PNEUMONA, PNEUMONIE, PNEUMONNIA, INFLUENZA, INFULENZA, INFLEUNZA, NFUENZA, FLU INFECTION, H1N1, HINI VIRAL, SWINEFLU, SWINEINFLU, SWINE FLU, SWINE INF, SMOKE INHALATION, PULMONARY FIBROSIS, CHEST INFECTION, CHEST SEPSIS, PNEUMOSEPSIS, PLEURAL EFFUSION, SHORTNESS OF BREATH, LOWER RESPIRATORY TRACT INFECTION, LOWER RESPIRATORY TRCAT INFECTION, INTERSTITIAL LUNG DISEASE, HYALINE MEMBRANE DISEASE, MACLEOD'S SYNDROME	
Cancer	METASTATIC, METASTASES, METASTASIS, MALIGNANT, MALIGNANCY, NEOPLASM, CANCER, CARCINOMA, TUMOUR, TUMOR, LEUKEMIA, LEUKAEMIA, LYMPHONA, HODGKIN, SECONDARIES, BLASTOMA, SARCOMA, GRANUCOMA	COMA, GLAUCOMA, STOMA, MYXOMA, HEMATOMA, HAEMATOMA, ATHEROMA, ASPERGILLOMA

2. Categorization of COVID-19 restrictions

A stringency index, categorized into five levels, was developed based on key restrictions implemented by the state government. Categorization of restrictions is described in detail elsewhere.[1] For the present paper, five levels of restrictions were used. Note that in all periods there were restrictions on international travel.

Level 1: Restrictions on large indoor and outdoor gatherings, including funerals, weddings and worship.

Level 2: No indoor gatherings; small outdoor gatherings permitted (varied).

Level 3: 4 reasons to leave home (grocery shopping, work if an essential worker, provision of receipt of health care, exercise).

Level 4: 4 reasons to leave home + masks, and, later, a 21:00 curfew.

Level 5: State of Disaster declared; 4 reasons to leave home + a 20:00 curfew; travel not permitted beyond a 5-km radius; masks required in indoor and outdoor areas.

3. R script for estimating excess mortality

Data are structured as shown below. The same format is used for each cause and each age group

```
head(df)
# week  week_start_date    year cause     count        ERP    wMR
# <dbl> <date>             <dbl>  <chr>   <int>       <dbl>  <dbl>
# 1     1 2015-01-04       2015 all_cause   759     6022322   12.6
# 2     1 2016-01-03       2016 all_cause   684     6173172   11.1
# 3     1 2017-01-01       2017 all_cause   752     6321606   11.9
# 4     1 2017-12-31       2018 all_cause   760     6462019   11.8
# 5     1 2018-12-30       2019 all_cause   708     6596880   10.7
# 6     1 2019-12-29       2020 all_cause   709     6729626.  10.5

# predict deaths
head(df)
all_cause <- df %>%
  mutate(sin_of_week_num = sin(2*week*pi/52.18),
         cos_of_week_num = cos(2*week*pi/52.18),
         week_num=as.numeric(week_start_date))

mod <- MASS::rlm(wMR~week_num + sin_of_week_num + cos_of_week_num,
                 data=subset(all_cause, year<2020), psi=MASS::psi.bisquare)

all_cause_emr <- cbind(all_cause,
                       predict(mod, all_cause, interval = "prediction"))

# to plot the predicted and observed rates
all_cause_emr %>%
  rename(Predicted="fit", Observed="wMR") %>%
  pivot_longer(cols=c("Predicted","Observed"),
               values_to = "rate", names_to = "rate_type") %>%
  arrange(week_start_date) %>%
  filter(year==2020) %>%
  ggplot(aes(x = week)) +
  geom_ribbon(aes(ymin=lwr, ymax=upr, fill="95%PI"), alpha=0.15) +
  geom_line(aes(y = rate, lty=rate_type)) +
  geom_vline(xintercept = 0, colour="#201547") +
  geom_hline(yintercept = 8, colour="#201547") +
  scale_x_continuous(limits = c(0,53.5),
                     breaks = seq(1,53,4),
                     expand = c(0,0)) +
  scale_y_continuous(limits=c(8,16),
                     breaks = seq(8,16,2),
                     labels = seq(8,16,2),
```

```
                     expand = c(0,0)) +
  labs(y="Estimated mortality rate per 100,000",
    x="Week", lty="Rate (year)", fill="") +
  theme_minimal() +
  theme(legend.position = c(0.19,0.85),
        legend.direction = "vertical", legend.box = "horizontal") +
  guides(linetype = guide_legend(order = 1), fill = guide_legend(order = 2))
```

References

1. Victorian Department of Health COVID-19 writing group. Population-based analysis of the epidemiological features of COVID-19 epidemics in Victoria, Australia, January 2020–March 2021, and their suppression through comprehensive control strategies. Lancet Reg Health West Pac. 2021;17:100297. doi:10.1016/j.lanwpc.2021.100297 pmid:34723232
